# Sleep Alterations in a Mouse Model of Spinocerebellar Ataxia Type 3

**DOI:** 10.3390/cells11193132

**Published:** 2022-10-05

**Authors:** Maria-Efstratia Tsimpanouli, Anjesh Ghimire, Anna J. Barget, Ridge Weston, Henry L. Paulson, Maria do Carmo Costa, Brendon O. Watson

**Affiliations:** 1 Department of Neurology, Michigan Medicine, University of Michigan, Ann Arbor, MI 48109, USA; 2 Department of Psychiatry, Michigan Medicine, University of Michigan, Ann Arbor, MI 48109, USA

**Keywords:** polyglutamine, ataxin-3, sleep, EEG, beta-oscillations

## Abstract

Spinocerebellar ataxia type 3 (SCA3) is a neurodegenerative disorder showing progressive neuronal loss in several brain areas and a broad spectrum of motor and non-motor symptoms, including ataxia and altered sleep. While sleep disturbances are known to play pathophysiologic roles in other neurodegenerative disorders, their impact on SCA3 is unknown. Using spectrographic measurements, we sought to quantitatively characterize sleep electroencephalography (EEG) in SCA3 transgenic mice with confirmed disease phenotype. We first measured motor phenotypes in 18–31-week-old homozygous SCA3 YACMJD84.2 mice and non-transgenic wild-type littermate mice during lights-on and lights-off periods. We next implanted electrodes to obtain 12-h (zeitgeber time 0-12) EEG recordings for three consecutive days when the mice were 26–36 weeks old. EEG-based spectroscopy showed that compared to wild-type littermates, SCA3 homozygous mice display: (i) increased duration of rapid-eye movement sleep (REM) and fragmentation in all sleep and wake states; (ii) higher beta power oscillations during REM and non-REM (NREM); and (iii) additional spectral power band alterations during REM and wake. Our data show that sleep architecture and EEG spectral power are dysregulated in homozygous SCA3 mice, indicating that common sleep-related etiologic factors may underlie mouse and human SCA3 phenotypes.

## 1. Introduction

Spinocerebellar ataxia type 3 (SCA3), also known as Machado-Joseph disease, is a fatal and incurable dominantly inherited ataxia with typical adult onset of neurodegenerative symptoms [1]. SCA3 is caused by a CAG repeat expansion in the ATXN3 gene encoding a polyglutamine stretch in the protein ataxin-3 (ATXN3) [2], which is ubiquitously expressed throughout the brain and the body [3,4]. People with SCA3 experience a wide variety of motor impairments and non-motor neurological symptoms, depending on the areas of the central and peripheral nervous system affected by the degenerative process [5,6,7,8]. The brain areas that are most frequently affected include the cerebellum, brainstem, cerebral cortex, basal ganglia, thalamus, and midbrain [5,6]. However, disease onset is determined based on the onset of progressive cerebellar ataxia, which is usually the main SCA3 symptom [9]. 

In SCA3, sleep disorders are common and have been identified in up to 60% of patients at early and later stages of disease [9,10]. In some SCA3 cases, sleep disorders precede ataxia onset by 5–10 years, implying a possible pathophysiologic role for sleep in this disease [11,12]. The most common sleep disorders in SCA3 are insomnia [13,14], excessive daytime sleepiness [15,16,17,18,19,20], restless leg syndrome [11,13,14,17,21,22,23,24,25,26], periodic limb movements disorder [15,17,23,27,28], REM sleep without atonia [23,27,28,29], REM sleep behavior disorder (RBD) [11,12,13,16,23,27,28,29,30,31,32], obstructive sleep apnea [13,15,18], confusional arousals [15,28], and sleep terrors [28,29]. However, we lack knowledge of sleep structure changes and electrophysiology in SCA3. Such knowledge, combined with details of which neuroanatomical regions are driving those changes, can enable mechanistic studies of the role of sleep in SCA3 progression, as has been implicated in other neurodegenerative diseases such as Huntington (HD), Parkinson (PD), and Alzheimer (AD) diseases. While anatomy–physiology correlates can be studied with spectrographic analysis of electroencephalogram (EEG) data during sleep, only one spectrographic sleep study of SCA1, 2 and 3 patients has been carried out and it only focused on sleep spindles without quantification of other oscillation frequencies [33]. 

Neurobiologic mechanistic studies are often most powerfully carried out in mouse models, given the molecular, genetic, and neuroscientific tools available in those models. Mouse models have been instrumental in revealing SCAs’ disease mechanisms. The SCA3 YACMJD84.2 (Q84) transgenic mouse, which expresses the full-length human disease ATXN3 gene, replicates many aspects of SCA3 including motor impairments and thus is frequently used in preclinical studies of SCA3 [34,35]. Homozygous mice (Q84/Q84) show motor impairments by six weeks of age [35]. The further use of these models in combination with powerful in vivo neurobiological tools can allow us to efficiently understand the impacts of this gene mutation on brain function states in wake and sleep. Despite the overall lack of spectrographic sleep studies in SCA3 mouse models, one previous study analyzing just 30 s of EEG identified increased energy in the alpha, beta, theta, and delta power bands in 12-month-old Q84 mice compared with wild-type mice [36]. Longer EEG recordings, encompassing distinct periods of sleep and wake, could provide quantitatively and qualitatively unique information about EEG dynamics in SCA3.

Given the sleep disorders reported in SCA3 patients and the above findings, we hypothesized that Q84 mice will show disrupted sleep structure and altered EEG spectral signatures during REM and NREM. Using 12-h EEG recordings and spectrographic analysis, we sought to determine whether sleep is indeed disturbed in the Q84 mouse model of SCA3, and to evaluate whether parietal and frontal neural oscillations are altered in these mice in specific sleep states.

## 2. Materials and Methods

### 2.1. Experimental Design

**Figure 1:**
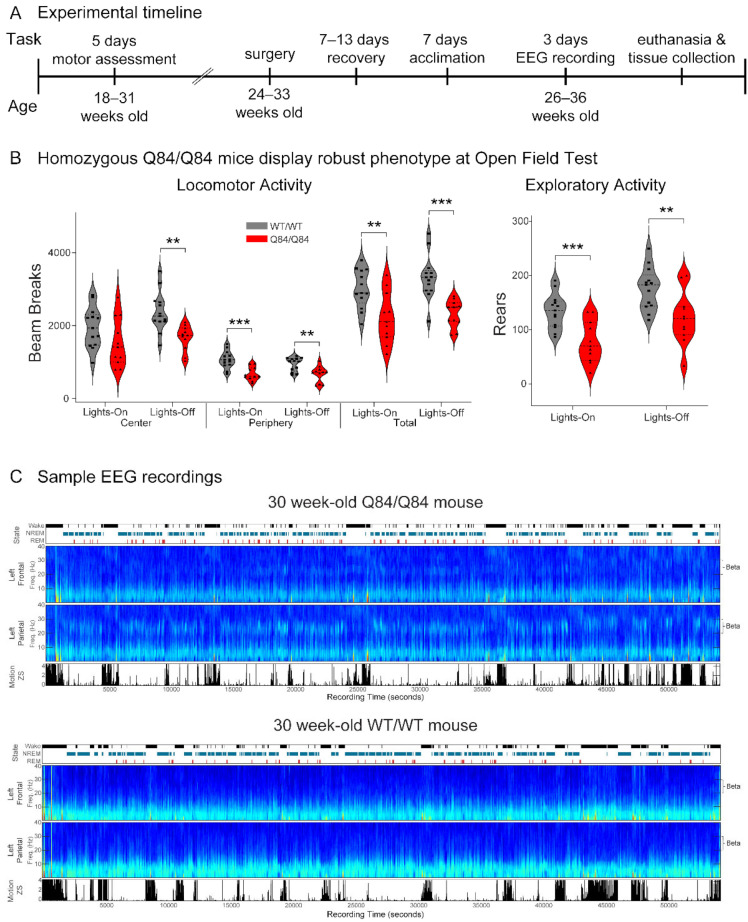
Measurement of motor behavior and sleep in SCA3 Q84 mice. (**A**) The motor behavior of 18–31-week-old mice was assessed in the morning with the lights on and in the evening with the lights off for five consecutive days. Next, mice underwent surgery to implant the recording electrodes at 24–33 weeks of age and, after 7–13 days of recovery, mice were acclimated to the recording equipment for one week. Finally, EEG recordings were obtained when the mice were 26–36-week-old for three consecutive days. (**B**) Homozygous Q84/Q84 mice (red, *n* = 11) display decreased locomotor (beam breaks) and exploratory (number of rears) activities on the open-field test for 30 min compared with wildtype WT/WT mice (grey, *n* = 13). Statistical analysis conducted according to repeated measures ANOVA with Bonferroni corrected post hoc estimated marginal means test, ** *p* < 0.01, *** *p* < 0.001. In violin plots, squares and triangles: individual values for wild-type and homozygous mice, dashed lines: median, dotted lines: quartiles. (**C**) Representative EEG recordings on the third day of evaluation of 30-week-old homozygous (top) and wild-type (bottom) mice. For each mouse, there is a display of the hypnogram (black: wake, blue: non-rapid-eye movement sleep (NREM), red: rapid-eye movement sleep (REM)), spectrograms of the left frontal and parietal electrodes, and movement variance. Spectrograms show frequency power versus time from EEG recordings and this data was analyzed to determine differences between genotypes in oscillatory power during each of wake, REM and NREM states. Increased beta power (20–30 Hz) can be seen in the homozygous mouse during REM and NREM.

We used Q84/Q84 and WT/WT littermates. Genotyping was performed by PCR on DNA isolated from tail biopsy at the time of weaning and post-mortem, as previously described [35]. Mice of both sexes were used in all experiments. There were no significant sex or age differences among genotypes at any time point (Table 1). As shown in our timeline (Figure 1A), first we weighed all mice and assessed motor function over five consecutive days to confirm the published phenotypic characteristics of Q84 mice [35] and to investigate circadian differences among genotypes. Following behavioral assessment, mice were split into groups of four for electrophysiology. Before surgery, mice were housed in cages with a maximum number of five animals, whereas post-surgery they were single housed. At all times mice were maintained in a standard 12-h light/dark cycle (zeitgeber time (ZT) 0/12) with food and water ad libitum. One group had 12 weeks to adjust to a one-hour forward time shift due to daylight savings time. For each group, stereotaxic craniotomy surgeries were performed over one week (details below), which were followed by periods of recovery and acclimation to the recording equipment. Electrophysiologic recordings were then performed for three consecutive days while the mice behaved and slept freely. We analyzed EEG data from the third recording day.

### 2.2. Motor Function Evaluation

Motor function was assessed in 18–31-week-old mice (Table 1), Q84/Q84 (*N* = 11; 5 females, 6 males) and WT/WT (*N* = 12; 5 females, 7 males), twice daily for five consecutive days using tests as previously described [35]. Each day, the first, lights-on, session started at approximately zeitgeber time (ZT) 3.5 and the second, lights-off, session started at ZT 15.5. During the lights-on sessions, the average light intensity in the experimental room was 75 lux. In lights-off sessions, mice were only exposed to red wavelength lights. During the first four consecutive days (Days 1–4) we assessed performance on round and square balance beams followed by the rotarod test. On Day 5, we evaluated locomotor and exploratory activities in an open field chamber for 30 min. 

For balance-beam testing, mice crossed a 44-cm-long plexiglass beam from an open clear platform to an enclosed black platform both placed at 53 cm of height [35] for two consecutive trials on each of the four consecutive days and nights. Mice crossed first a 1 cm diameter round beam, and then a 0.5 cm-wide square beam. Time to traverse the beam was recorded for each trial with a 20-s maximum cutoff, and falls were scored as 20 s. 

For rotarod testing, mice were placed on a rod accelerating from 4 to 40 revolutions per minute (RPM) over the course of 300 s (ENV-574M, Med Associates Inc.). Each animal performed two trials in the lights-on session and two trials in the lights-off session for four consecutive days. Mice rested for at least 30 min between the two trials. For each trial, the latency to fall off the rod was recorded, and was recorded as 300 s for animals that did not fall. 

For the open field test [35], each animal was placed once in the lights-on session and once in the lights-off session in the center of an open-field apparatus with XYZ infrared beams (San Diego Instruments, San Diego, CA, USA). The number of beam brakes (locomotor activity) and rears (exploratory activity) was recorded for 30 min.

### 2.3. Electrophysiology

Surgeries were performed on 24–33-week-old mice after testing for motor performance. We induced anesthesia with 4% isoflurane, administered carprofen IP for analgesia, and then placed the mouse in a stereotaxic frame, maintaining 0.5–2% isoflurane-induced anesthesia via nose cone. We also administered bupivacaine along the scalp midline before incision. After scalp incision, we drilled six holes, two for the frontal electrodes at +2 anterior/posterior (AP) and +/−1.25 medial/lateral (ML), two for the parietal electrodes at +/-2 AP and +/−1.25 ML, and two on the cerebellum, just below the lambda at +/−1.25 ML. In the holes, we placed 4–5 mm screws with already soldered wires. These wires were then soldered to a 32 or 64 channel Omnetics connector. The assembly was fixed to the screws and skull using acrylic dental cement, Unifast Trad. A copper mesh cage was then placed around the assembly. The cerebellar electrode at +1.25 ML was soldered to the copper cage to act as ground. After two Q84/Q84 mice died shortly after the surgery, we administered intraperitoneally methylprednisolone (30 mg/kg) to the rest of the mice to increase survival rate. Overall, we observed increased mortality in transgenic mice post-surgery that may relate to the previously observed genotype-based sensitivity [35]. After recovering for 7–13 days following surgery, mice were acclimated to their individual boxes in the recording room and were plugged in to the EEG recording equipment daily for one week, starting from 1 h and gradually increasing to 10 h.

We successfully obtained EEG recordings from 26–36-week-old Q84/Q84 (*N* = 5; 3 females, 2 males) and WT/WT mice (*N* = 9; 5 females, 4 males) (Table 1). EEG recordings were collected for three consecutive days in boxes grounded using aluminum foil. The first two days served as further acclimation to the recording procedure and thus were not analyzed. Daily, mice were transferred from the housing room to the recording room, and while the lights were still off, each mouse was placed into its recording box. Recordings started about 10 min before the lights were on and stopped about three hours after lights were off. Recordings were performed at 20,000 Hz using an Intan RHD2000 Evaluation board and a preamplifier with an accelerometer chip to record movement in three dimensions. Mice were allowed to sleep and behave freely during these sessions. We recorded up to four mice at the same time.

### 2.4. Sleep and EEG Analysis

Sleep scoring was performed by an automated MATLAB algorithm (SleepScoreMaster.m, accessed on 7 July 2021) and manually checked (TheStateEditor.m, accessed on 7 July 2021) from the ‘buzcode’ library (https://github.com/buzsakilab/buzcode, accessed on 7 July 2021) as in previous work [37]. Two WT/WT mice had one electrode each (one left parietal and one left frontal) excluded from all analyses due to lack of signal. Spectrographic power, theta ratio and slow-wave activity, and movement estimates, based on the variance of the accelerometer sensors, were used to segregate REM, NREM, and wake brain states. Further analysis was done using custom code in MATLAB.

Here, we analyzed EEG data from the third day of recordings, when the lights were on and mice typically spend more time sleeping. In addition to the total amount of time spent in each brain state, we also assessed sleep fragmentation by counting the number of bouts for each state and calculating their average duration. For spectral power analysis, we excluded extreme outliers separately for each channel by removing any seconds with elements that were more than 15 interquartile ranges above the upper quartile or below the lower quartile. Next, the power data for each frequency band of interest was normalized by dividing over the median value in that band over the 12-h lights-on period. Before quantification, the power bands were defined as: delta 0.5–4 Hz, theta 5–10 Hz, spindle 11–19 Hz, beta 20–30 Hz, gamma 40–100 Hz, and ripple 130–180 Hz. Finally, to create average spectra we calculated the mean normalized spectral power per band for each brain state in the frontal and parietal electrodes.

### 2.5. Statistics

Statistical analysis of the behavioral tests was performed on SPSS using repeated measures ANOVAs and post hoc estimated marginal means, with day, time of day, and trial, where appropriate, as within-subjects factors and genotype as between-subjects factor. To assess age, sleep stage metrics and spectral power differences between genotypes we used independent *t*-test or Mann–Whitney test, depending on whether the data were normally distributed and had equal variances or not. Chi-square test was used to assess whether sex ratios differed among genotypes. For all tests, the level of significance was determined as *p* < 0.05. All post hoc tests were Bonferroni corrected.

## 3. Results

### 3.1. SCA3 Mice Show Similar Motor Impairment in the Lights-on Phase to Previous Findings

We tested SCA3 Q84 mice for five consecutive days and nights at 18–31 weeks of age, when overt motor impairment has been previously reported [35]. First, we confirmed that Q84/Q84 mice replicated previous findings [35] in loss of weight gain and motor impairments evaluated in the lights-on phase across five days (Figure 1 and Figure S1). We found a statistically significant main effect of the genotype in the round balance beam performance and all measures of open field behavior but not in the square balance beam or the rotarod (Figure 1B and Figure S1). Q84/Q84 mice were slower to traverse the round balance beam (Figure S1A) and displayed fewer beam breaks and rears in the open field test (Figure 1B) than WT/WT mice. Moreover, in some trials on the first two days of motor assessment, Q84/Q84 mice were slower to traverse the square balance beam and fell faster from the rotarod than WT/WT mice (Figure S1B and C). Overall, while the 18–31-week-old homozygous Q84/Q84 mice used in this study showed robust loss of weight gain and motor dysfunction. 

### 3.2. SCA3 Mice Show a Trend for Circadian Differences

Given the lack of previous circadian studies of motor behavior in SCA3 mouse models, here we investigated movement symptoms in SCA3 Q84 transgenic mice during both light and dark phases of the 24-h cycle. In line with the nocturnal nature of lab mice, we found a main effect of the time of the day (lights-on or off) in both balance beams, the rotarod, locomotor activity in the central area of the open field, and exploratory activity, with all the mice being more active during the lights-off sessions (Figure 1B). Whereas we did not observe any overall statistically significant interactions between genotype and time of the day, post hoc tests indicated some circadian differences for each genotype (Figure 1, Figure S1): (1) only the Q84/Q84 mice traversed the round balance beam faster in the lights-off session than in the lights-on session(*p* < 0.05); (2) in contrast, only the WT/WT mice traversed the square balance beam faster (*p* < 0.05), stayed longer on the rotarod (*p* < 0.001) and had more beam breaks in the central area of the open-field (*p* < 0.01) during the lights-off session in comparison to the lights-on session; and (3) both genotypes demonstrated higher exploratory activity during the lights-off session compared to the lights-on session (both *p* < 0.01).

### 3.3. SCA3 Mice Display Increased REM Duration and Sleep/Wake Fragmentation Compared to Wild-Type Mice

**Figure 2:**
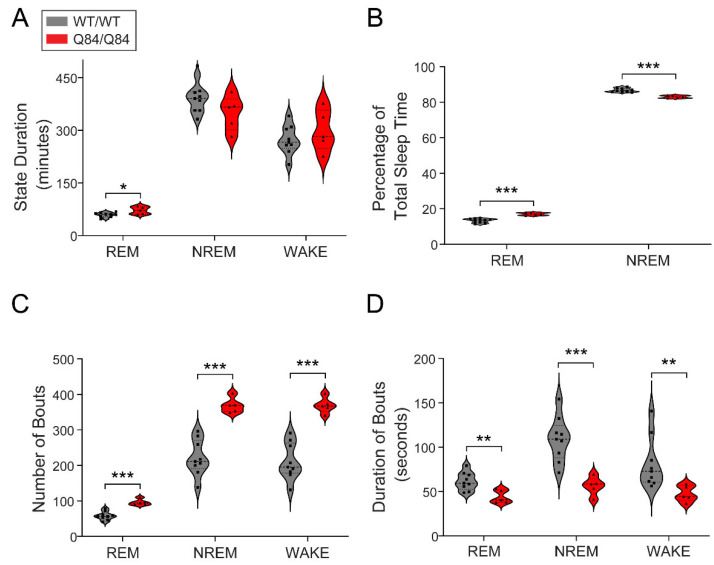
SCA3 Q84 mice show increased REM duration and sleep/wake fragmentation. (**A**) Homozygous Q84/Q84 mice (red, *n* = 5) reveal longer REM duration overall than wild-type WT/WT (grey, *n* = 9) littermates. (**B**) Homozygous mice show increased REM and decreased NREM percentages of total sleep time (TST) in comparison to wild-type mice. (**C**) Homozygous mice show more bouts of each state than their wild-type littermates. (**D**) In all states, homozygous mice display shorter bouts than wild-type mice. Thus, homozygous mice show that both sleep and wake states are composed of more bouts of shorter duration than wild-type mice. All violin plots display results from the 12 h lights-on period (zeitgeber time (ZT) 0–12), squares and triangles: individual values for wild-type and homozygous mice, dashed lines: median, dotted lines: quartiles. Statistical difference according to independent *t*-test or Mann–Whitney test. * *p* < 0.05, ** *p* < 0.01, *** *p* < 0.001, REM: rapid-eye movement sleep, NREM: non-REM.

After examining the circadian aspects of the motor phenotype, we studied the impact of the ATXN3 CAG expansion on sleep in Q84 mice by conducting EEG recordings for three consecutive days in 26–36-week-old mice continuously for 12 h during the lights-on period, when mice typically sleep. We used software with manual verification to score each one-second period of the third recording day as either REM, NREM or wake. In terms of total duration of each state, we only observed an increase in total REM duration in Q84/Q84 mice compared with WT/WT mice (Figure 2A). We also found that Q84/Q84 mice had higher REM and lower NREM as percentages of total sleep time compared with WT/WT mice (Figure 2B). Importantly, we found that Q84/Q84 mice showed an increased number of bouts and shorter bout duration of REM, NREM, and WAKE compared to WT/WT mice, all *p* < 0.05 (Figure 2C and D), indicating increased sleep fragmentation in these mutant mice, similar to previous observations in SCA3 subjects [28,29,38].

### 3.4. SCA3 Mice Display Altered Neural Oscillations during REM and NREM Sleep

**Figure 3:**
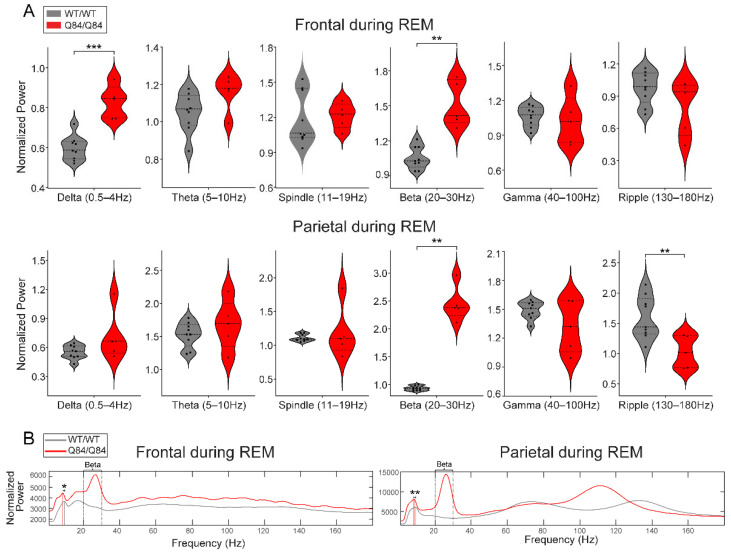
In REM sleep, homozygous Q84/Q84 mice show altered oscillatory power across many frequency bands, most clearly in frontal sites. (**A**) Homozygous Q84/Q84 mice (red, *n* = 5) show higher oscillatory power compared to the wild-type WT/WT (grey, *n* = 9littermates in the delta and beta frequency bands in frontal sites during REM. In parietal sites during REM, homozygous mice show higher beta and lower ripple activity than the wild-type mice. In violin plots, squares and triangles represent individual values for wild-type and homozygous mice, dashed lines display the median, and dotted lines show quartiles. (**B**) The total power averaged across all seconds of REM for each genotype plotted for better visualization. Homozygous mice show higher power across the low frequency bands including delta, theta, spindle, and beta bands spanning from 0.5–30 Hz in both frontal and parietal electrodes. Vertical grey and red lines indicate the peak theta frequency for wild-type and homozygous mice. In the frontal and parietal sites, theta peak is slower in the homozygous mice than in the wild-type mice. Frontal sites show higher power in all frequencies in fact, but parietal sites do not show overall changes in gamma power, rather only a frequency shift of a power bump from around 135 Hz to 110 Hz. All plots display results from the 12 h lights-on period (zeitgeber time (ZT) 0–12) and power is normalized by dividing over the respective frequency’s median value. Statistical difference only according to independent *t*-test or Mann–Whitney test. ** *p* < 0.01, *** *p* < 0.001. REM: rapid-eye movement sleep.

**Figure 4:**
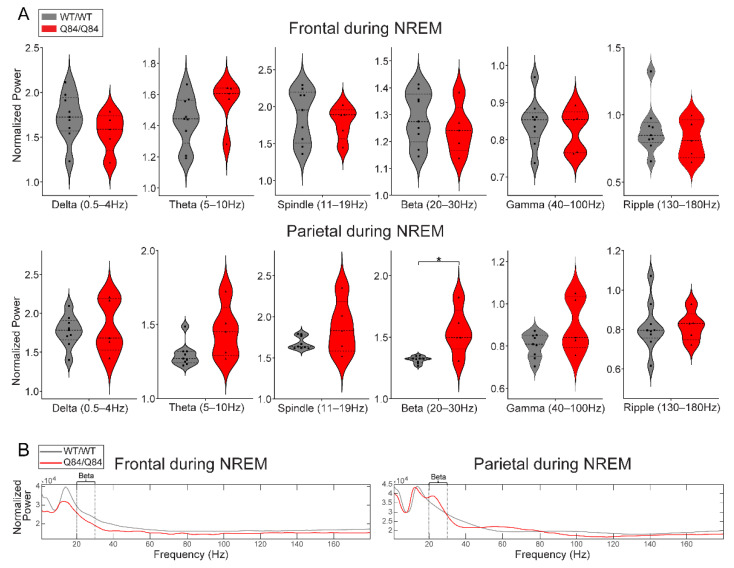
In NREM sleep, homozygous Q84/Q84 mice show increased parietal beta power. (**A**) Homozygous Q84/Q84 mice (red, *n* = 5) show higher oscillatory power compared to the wild-type WT/WT (grey, *n* = 9) in the parietal beta band power during NREM. In violin plots, squares and triangles represent individual values for wild-type and homozygous mice, dashed lines display the median, and dotted lines show quartiles. (**B**) The total power averaged across all seconds of NREM for each genotype is plotted for better visualization. Trends towards reduced delta and spindle power can be appreciated – both thalamocortical rhythms. All plots display results that come from the 12 h lights-on period (zeitgeber time (ZT) 0–12) and power is normalized by dividing over the respective frequency’s median value. Statistical difference according to independent *t*-test or Mann–Whitney test. (red: homozygous Q84/Q84 mice (*n* = 5), grey: wild-type WT/WT mice (*n* = 9)) NREM: non-rapid-eye movement sleep, * *p* < 0.05.

**Figure 5:**
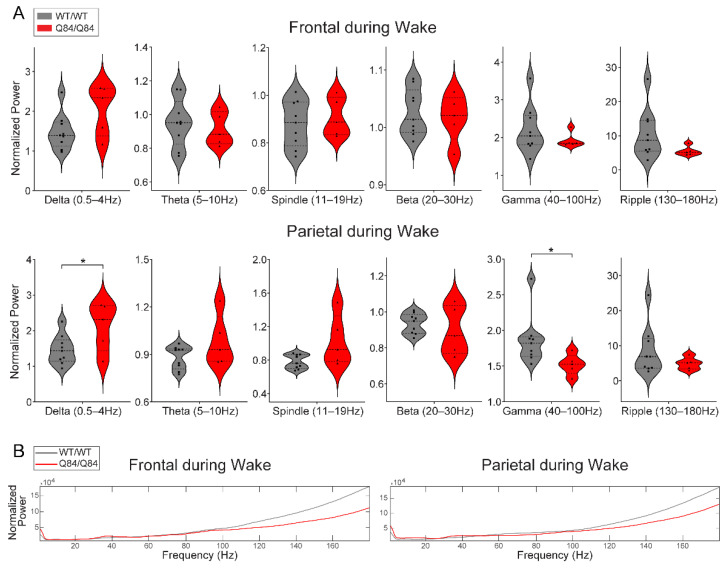
In wake, homozygous Q84/Q84 mice show decreased parietal gamma power. (**A**) Homozygous Q84/Q84 mice (red, *n* = 5) show increased delta and decreased gamma power in the parietal area during wake when compared to their wild-type WT/WT (grey, *n* = 9) littermates during wake. In violin plots, squares and triangles represent individual values for wild-type and homozygous mice, dashed lines display the median, and dotted lines show quartiles. (**B**) The total power averaged across all seconds of wake for each genotype is plotted for better visualization. Normalization by dividing over the total median value across the full recording was kept to allow for consistency with data in part A, but that creates the upward trend towards higher frequency bands. All plots display results that come from the 12 h lights-on period (zeitgeber time (ZT) 0–12) and power is normalized by dividing over the respective frequency’s median value. Statistical difference according to independent *t*-test or Mann–Whitney test. Significance as * *p* < 0.05.

Many movement disorders, including the synucleinopathies, display altered oscillatory states during sleep relative to healthy subjects, even preceding clinical disease onset [39,40]. To quantify differences in brain EEG oscillations between SCA3 mice and controls, we used spectrographic analysis to quantify the power of all EEG oscillations across sleep (REM and NREM) and wake states (Figure 3, Figure 4 and Figure 5). Visual examination of the spectrograms of all Q84/Q84 mice revealed the presence of beta power during REM and NREM (Figure 1C).

First, we validated our visual finding of increased beta power in Q84/Q84 mice compared to WT/WT mice during REM and NREM (Figure 3A and Figure 4A): whereas during REM, beta power was increased in both frontal and parietal regions (Figure 3A), during NREM it was only increased in the parietal area (with significance set at *p* < 0.05 by Kruskal–Wallis) (Figure 4A).

We verified additional cross-genotype anatomy-specific alterations of brain oscillations during REM. During REM, compared with WT/WT mice the Q84/Q84 mice revealed higher frontal delta power (Figure 3A), lower parietal ripple power (Figure 3A), and slower peak frequency of theta rhythm in both frontal and parietal areas (Figure 3B). Finally, during wake, we observed higher delta and lower gamma oscillations in the parietal area of Q84/Q84 mice compared to WT/WT littermates (Figure 5A).

## 4. Discussion

In this study, we sought to identify alterations in sleep architecture and EEG spectral power in motor impaired SCA3 Q84 transgenic mice that are frequently used in preclinical trials for SCA3 [35]. EEG analysis of 26–36-week-old homozygous Q84 mice and wild-type littermates allowed us to identify quantitative sleep changes in these mice: (a) increased fragmentation in all states and REM duration; (b) higher beta power during REM and NREM; and (c) several alterations of brain EEG oscillations during REM and wake including in the delta and gamma bands. Importantly, both Q84 mice and patients with SCA3 have alterations in REM and NREM sleep.

As expected, 18–31-week-old Q84/Q84 mice displayed motor impairments and reduced weight gain. We also report circadian differences between genotypes, as in some motor tasks only one genotype shows more motor activity in the lights-off session than the lights-on sessions. These novel circadian findings in our work may be partially due to the difficulty of each task in combination with the degree of motor impairment in the Q84/Q84 mice, and/or due to learning defects.

In Q84/Q84 mice, we found that sleep architecture displayed increased fragmentation in REM, NREM, and wake periods. Importantly, this mirrors findings of sleep fragmentation [15,29,41] and decreased sleep efficiency [29] previously identified in SCA3 patients. Such sleep fragmentation can disrupt the homeostatic functions of sleep and thus have a direct impact on proper neurobiological function, including impairment of protein homeostasis and decreased clearance of toxic proteins [42,43]. The detrimental effects of chronic sleep fragmentation were observed in an AD mouse model where sleep fragmentation increased amyloid β deposition [44]. The sleep fragmentation we observed seems to be a common feature in neurodegenerative diseases [45,46,47,48,49,50,51,52] and could, therefore, contribute to improper clearance of the misfolded and aggregation-prone mutant ATXN3 protein in SCA3.

We found an overall increase in REM in Q84/Q84 mice, both in duration and as percentage of total sleep time. At the same time, we observed an increase in the number of REM bouts and a decrease in the duration of the individual sleep bouts. In SCA3 patients, REM findings are mixed. One study (*n* = 15) showed decreased REM duration [29], but a larger study (*n* = 47) showed no changes in REM duration [28]. Patients in both studies had similar age at the evaluation time, age at disease onset, and size of the expanded CAG repeat but different numbers of patients and sex ratios. Overall, REM architecture appears to be affected by SCA3. How REM is dysregulated may depend on which areas of the brainstem and cerebellum are affected by SCA3 neuronal loss [5,6] since they both contain circuits controlling REM [53,54,55,56,57]. Age may also play a role in this progressive disease, and longitudinal imaging and sleep studies assessing REM at different disease stages in individuals with SCA3 and SCA3 animal models are needed.

While beta-band changes were most prominent during REM, we found additional EEG alterations of delta and theta band powers and theta band frequency. These results are similar to previous EEG findings based on 30 s recordings in 12-month-old SCA3 Q84 mice [36], although it is unclear in which alertness state these findings occurred. We also observed a decrease in ripple power during REM and an increase in gamma power during wake in homozygous mice. While there have been findings of decreased spindle (11–16 Hz) density in SCA3 patients [33], in our 6.5–9-month-old SCA3 Q84 mouse model we did not observe any significant differences in spindle power (11–19 Hz).

Most prominently, we showed increased beta power during NREM and REM sleep in Q84 mice. In Q84/Q84 mice, higher beta power was seen across all anatomical locations during REM but only in the parietal electrodes during NREM. Increased beta in the motor cortex has been reported before in this SCA3 mouse model [36], although it is unclear in which sleep state. Based on mouse brain anatomy [58,59], the parietal electrodes are closest to the basal ganglia and thus more likely to capture signals from that area than the frontal electrodes. This is significant since the basal ganglia are known to be affected by neuronal loss and ATXN3 aggregation in SCA3 patients [6]. The increased beta power during REM and NREM in Q84 mice is reminiscent of increased beta oscillations observed in other neurodegenerative diseases and sleep disorders. Alterations, most frequently increases, in beta oscillations have been observed during wake and sleep states in patients or animal models of PD [60,61,62,63,64,65,66,67,68], HD [69,70,71,72,73,74,75,76,77,78,79,80,81,82,83,84], dementia with Lewy bodies [85,86], insomnia [60,87], and RBD [63,88,89,90]. In PD, beta oscillations in the basal ganglia are usually pathological [60,62,64,67,68,91] and appear to be linked to basal ganglia dysfunction or compensation. As shown both in our results and by the discussed similarities to other motor deficit conditions, increased beta oscillations may reflect changes in the basal ganglia circuits that persist even during sleep. Thus, the triad of RBD, beta oscillations, and basal ganglia alterations appears to be a unifying feature of these motor diseases, including SCA3.

Clinically, RBD is considered a predictor of some neurodegenerative disorders, including synucleinopathies and dementias [92,93]. It is known that SCA3 patients experience RBD and other sleep changes [94]. Moreover, beta event-related synchronization at a motor task appears to be reduced in SCA3 patients in comparison to healthy controls [95], but it is yet to be determined whether SCA3 patients also show increased beta activity during REM. These findings indicate that the combination of RBD and basal ganglia-based beta-oscillatory changes could represent an overlap in meso-scale pathology across SCA3 and other neurodegenerative disorders. This change may represent a primary alteration or compensation for other pathology.

In summary, we found that sleep architecture and specific brain oscillations, in particular beta power, are altered in motor impaired SCA3 Q84 mice. The sleep EEG abnormalities identified here may point to altered neuronal circuits pertinent to SCA3, such as the basal ganglia, yielding greater mechanistic detail of SCA3 pathogenesis, and should prompt additional human studies of spectral changes both in and out of sleep. Many of our EEG spectral findings were both state and anatomy-specific; accordingly, future deep-tissue recordings would be needed to better understand the anatomical and spiking implications of the observed regional alterations in SCA3 mice. Limitations here include (1) possible discrepancies in REM duration findings in mice versus humans, though currently reported human results are mixed; and (2) inability to assess sex and age effects in sleep EEG metrics in SCA3 mice given the low number of mice per subgroup. Longitudinal sleep EEG studies throughout the lifespan of people with SCA3 and transgenic mouse models will be needed to extend our observations on sleep architecture and spectral alterations, determine their relation to the progression of motor symptoms and brain pathophysiology, and evaluate their potential translational utility. Hence, specific quantifiable sleep and EEG changes may serve as a target for the development of disease-modifying therapies and as disease biomarkers in SCA3. 

## Figures and Tables

**Table 1:** Sex and age of homozygous Q84/Q84 and wild-type WT/WT mice used for motor assessment and EEG recordings.

Assessment Type	Genotype	Females (N)	Males (N)	Sex Differences	Min Age (Weeks)	Max Age (Weeks)	Mean Age (Weeks)	Age Differences
Motor Phenotype	Q84/Q84	5	6	Q84/Q84 vs. WT/WT: *p* > 0.05	17.71	31.14	24.55	Q84/Q84 vs. WT/WT: *p* > 0.05
	WT/WT	5	7		19.86	31.14	24.36	
EEG Recordings	Q84/Q84	3	2	Q84/Q84 vs. WT/WT: *p* > 0.05	30.14	35.86	31.80	Q84/Q84 vs. WT/WT: *p* > 0.05
	WT/WT	5	4		26.43	36.14	31.67	

All the mice for which EEG recordings were carried out were previously evaluated for motor performance. Sex and age differences were, respectively, evaluated by the Chi-square test and Mann–Whitney test. Significance was set at *p* < 0.05.

## Data Availability

The data that support the findings of this study are available upon request from the corresponding authors.

## Supplementary Materials

The following supporting information can be downloaded at: https://www.mdpi.com/article/10.3390/cells11193132/s1, Figure S1: Motor impairment and decreased weight in homozygous Q84/Q84 mice.
