# Supplementary material for: Sleep Alterations in a Mouse Model of Spinocerebellar Ataxia Type 3
Source: Cells. 2022 Oct 5;11(19):3132. doi: 10.3390/cells11193132 (PMC9563426; doi:10.3390/cells11193132)
Supplement: Supplementary file 1 [file cells-11-03132-s001.zip › cells-1858256-supplementary.pdf]

Supplementary Materials

Sleep alterations in a mouse model of Spinocerebellar ataxia type 3

Maria-Efstratia Tsimpanouli, Anjesh Ghimire, Anna J. Barget, Ridge Weston, Henry L. Paulson, Maria do Carmo Costa, Brendon O. Watson

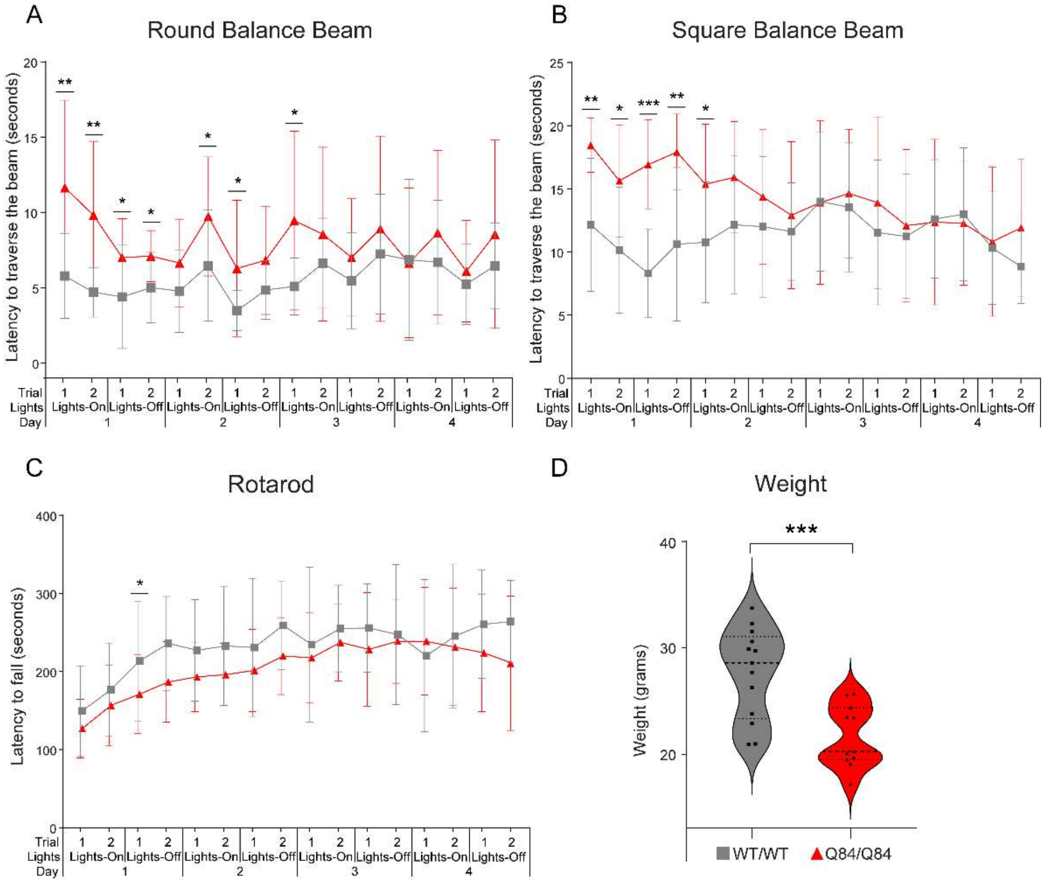

Figure S1. Motor impairment and decreased weight in homozygous Q84/Q84 mice. Comparison of phenotypic features in homozygous Q84/Q84 (red triangles, n=11) and wild-type WT/WT (grey squares, n=13) littermates: (**A**) Motor impairments in the round balance beam on the first three days of assessments; (**B**) Motor impairments in the square balance beam on the first two days of assessments; (**C**) Minor motor impairments in the accelerating rotarod on the first day of assessments; (**D**) Homozygous mice show a decrease in body weight compared with wild-type and hemizygous littermates. All motor tasks statistics are according to post hoc Bonferroni corrected estimated marginal means tests of repeated measures ANOVAs. Weight statistics according to independent t-test. Significance as * $p<0.05$, ** $p <0.01$, *** $p<0.001$.
